# Taxol-mediated augmentation of CD95 ligand-induced apoptosis of human malignant glioma cells: association with bcl-2 phosphorylation but neither activation of p53 nor G2/M cell cycle arrest.

**DOI:** 10.1038/bjc.1998.64

**Published:** 1998

**Authors:** W. Roth, B. Wagenknecht, C. Grimmel, J. Dichgans, M. Weller

**Affiliations:** Department of Neurology, University of TÃ¼bingen, School of Medicine, Germany.

## Abstract

**Images:**


British Journal of Cancer (1998) 77(3), 404–411
© 1998 Cancer Research Campaign

### Taxol-mediated augmentation of CD95 ligand-induced apoptosis of human malignant glioma cells: association with bcl-2 phosphorylation but neither activation of p53 nor $G_2$/M cell cycle arrest

**W Roth, B Wagenknecht, C Grimmel, J Dichgans and M Weller**

Laboratory of Molecular Neuro-Oncology, Department of Neurology, University of Tübingen, School of Medicine, Hoppe-Seyler-Strasse 3, 72076 Tübingen, Germany

**Summary** The anti-tumour alkaloid taxol shows strong cytotoxic and antiproliferative activity in two human malignant glioma cell lines, T98G and LN-229. CD95 (Fas/APO-1) ligand is a novel cytotoxic cytokine of the tumour necrosis factor (TNF) family that exerts prominent antiglioma activity. At clinically relevant taxol concentrations of 5–100 nM, taxol and CD95 ligand showed significant synergistic cytotoxicity and growth inhibition. High concentrations of taxol induced $G_2$/M cell cycle arrest in both cell lines. The synergy of taxol and CD95 ligand was independent of cell cycle effects of taxol as synergy was achieved at much lower taxol concentrations than $G_2$/M arrest and as cell cycle effects of taxol were unaffected by co-exposure to CD95 ligand. Similarly, high concentrations of taxol were required to induce p53 activity in the p53 wild-type cell line LN-229. This effect was not modulated by CD95 ligand, suggesting that synergy is also independent of p53 activation. However, taxol induced a mobility shift of the bcl-2 protein on immunoblot analysis, indicative of bcl-2 phosphorylation. Bcl-2 phosphorylation on serine was confirmed by immunoprecipitation and phosphoserine immunoblot analysis. Considering (1) that phosphorylation of bcl-2 interferes with its heterodimerization with bax and (2) the inhibition of CD95-mediated apoptosis by bcl-2, we propose that taxol sensitizes malignant glioma cells to CD95 ligand by increasing the functional bax/bcl-2 rheostat in favour of bax and thus cell death.

**Keywords**: malignant glioma; CD95 (Fas/APO-1); taxol; bcl-2; p53; synergy

The median survival of malignant glioma patients receiving cytoreductive surgery, radiotherapy and adjuvant chemotherapy does not exceed 1 year. There is thus an urgent need for new experimental approaches to effective forms of treatment and for the exploration of novel antineoplastic drugs.

Taxol is a plant-derived cytotoxic agent that has been proposed to act via a novel mechanism of action: prevention of tubulin depolymerization, stabilization of microtubules and promotion of tubulin polymerization (Schiff et al, 1979; Horwitz et al, 1986). The p53 tumour suppressor gene product mediates growth arrest and DNA repair or apoptosis in response to genotoxic stimuli. Its major effects depend on the activation of target genes, such as *p21*, associated with growth arrest, and *bax*, linked to p53-mediated apoptosis. Actions of taxol documented in vitro include induction of $G_2$/M cell cycle arrest in human leukaemic cells (Horwitz et al, 1986; Rowinsky et al, 1988), induction of p53 activity and expression of p21 (Blagosklonny et al, 1995), and induction of apoptosis in breast cancer and myeloid leukaemia cells (Bhalla et al, 1993; Blagosklonny et al, 1996). Selective disruption of p53 in otherwise genetically intact fibroblasts enhanced rather than decreased sensitivity to taxol (Wahl et al, 1996). Taxol-mediated antineoplastic effects on various transplanted tumors in vivo correlate with taxol-induced apoptosis but not with mitotic cell cycle arrest after treatment with taxol (Milross et al, 1996). More recently, it has been proposed that taxol may act to phosphorylate the bcl-2 protein and thereby attenuate bcl-2 affinity for the bax protein. Bax would mediate apoptosis when released from bcl-2 heterodimers (Blagosklonny et al, 1996; Haldar et al, 1996).

TNF-α, a pleiotropic cytokine mediating inflammatory and immunological reactions, has been demonstrated to act synergistically with taxol on ovarian carcinoma cells in vitro (Williams et al, 1992; Berkova and Pagé, 1995). However, TNF-α also decreases TNF-α receptor density in macrophages (Ding et al, 1990).

Taxol is used mainly for patients with platin-refractory ovarian cancer and breast cancer. Based on promising studies on the effects of taxol on cultured glioma cells (Cahan et al, 1994; Silbergeld et al, 1995) and glioma xenografts (Riondel et al, 1992), several clinical studies of taxol for human malignant glioma patients have been conducted (Chamberlain and Kormanik, 1995; Glantz et al, 1995*a*; Prados et al, 1996). The disappointing results of these studies are probably partly due to the poor penetration of taxol into the brain at doses that are tolerated when given systemically (Glantz et al, 1995*b*). Although taxol may penetrate pathological vessel walls within tumour tissue much more efficiently than intact brain vessel walls (Heimans et al, 1994), it is clearly desirable to expose as many tumour cells to taxol as long as possibly achievable. Using local controlled delivery devices, such as taxol polymers, may be useful in circumventing this problem (Walter et al, 1994). This local application might also prove useful combined with post-operative radiotherapy as taxol is a radiosensitizer for glioma cells in vitro (Tishler et al, 1992). We have been involved in the development of a novel approach to malignant glioma that is based on the targeted induction of apoptosis via activation of the CD95 cytokine

*Received 13 December 1996*
*Revised 10 June 1997*
*Accepted 8 July 1997*

*Correspondence to:* M Weller receptor protein (Weller et al, 1994, 1995*a–c*; Roth et al, 1997). The present study was designed to examine possible synergistic interactions between taxol, TNF-α and CD95 ligand to design an efficient immunochemotherapy for malignant glioma. Further, we have examined possible links between synergy, CD95 expression, cell cycle effects and activation of p53, p21, bax and bcl-2.

### MATERIALS AND METHODS

### Chemicals and cell lines

The human malignant glioma cell line LN-229 was kindly provided by Dr N de Tribolet (Lausanne, Switzerland). T98G human glioma cells were obtained from ATCC (Rockville, MD, USA). TNF-α was purchased from Boehringer Mannheim (Germany). p53 antibody pAb1801 was from Oncogene Science (Uniondale, NY, USA), human bcl-2 antibody from Dakopatts (Glostrup, Denmark) and phosphoserine antibody from Sigma (St Louis, MO, USA). p21 and bax antibodies were obtained from Santacruz (Santacruz, CA, USA). Trypan blue was purchased from Biochrom KG (Berlin, Germany), taxol (paclitaxel) and propidium iodide were from Sigma. FITC-UB2 anti-Fas antibody was from Immunotech (Hamburg, Germany), FITC-mouse IgG1 was from Sigma. The murine neuroblastoma cell line Neuro-2A was maintained in minimal essential medium (MEM) supplemented with 10% fetal calf serum (FCS), 2 mM glutamine, 1% non-essential amino acids and 1% penicillin/streptomycin. Neuro-2A cells engineered to produce soluble murine CD95 ligand have been described elsewhere (Rensing-Ehl et al, 1995). One unit of cytotoxic activity attributable to CD95 ligand in Neuro-2A supernatants was defined as the activity required for half-maximal killing of the CD95 antibody-sensitive glioma cell line LN-18 (Weller et al, 1994). The experiments using CD95 ligand-containing supernatants were performed using supernatant from pooled *neo* vector control Neuro-2A cells as control.

### Cell culture and detection of cytotoxicity and growth inhibition

The human glioma cell lines were cultured in Dulbecco's modified Eagle medium (DMEM) containing 10% FCS, 1 mM glutamine and 1% penicillin/streptomycin as previously described (Weller et al, 1994, 1995*b*). Viability and proliferation were assessed by crystal violet staining or by trypan blue dye exclusion. For cytotoxicity assays, $5 \times 10^3$ glioma cells were plated in 96-well plates, adhered for 24 h, exposed to taxol or CD95 ligand or TNF-α, or combinations thereof, for 72 h in complete medium. For growth inhibition assays, $1.5 \times 10^3$ glioma cells were plated in 96-well plates, adhered for 24 h, exposed to the respective agents, and allowed to recover for five generation times in fresh agent-free medium. Generation times were 24 h for LN-229 and 26 h for T98G.

### Flow cytometry

For cell cycle analysis, the glioma cells were exposed to taxol or CD95 ligand or both for 24 h, washed, incubated with trypsin for 3 min at 37°C, harvested, washed and fixed with 70% ice-cold ethanol. Approximately $10^6$ cells per condition were stained with propidium iodide (50 μg $ml^{-1}$ in phosphate-buffered saline (PBS), containing 100 μg $ml^{-1}$ RNAase A), washed and subjected to flow cytometric analysis of DNA content using a Becton Dickinson FACScalibur cytometer. Results are presented as histograms. The percentage of apoptotic cells after exposure to taxol or CD95 ligand or both was also assessed by FACS analysis. After treatment, the cells were harvested by trypsinization, washed with Dulbecco's PBS and fixed with ice-cold 70% ethanol. The dead cells of each sample, which had detached from the culture flask, were harvested from the supernatant by centrifugation and subjected to the same procedure as the non-detached (viable) cells. One day after fixation the cells were centrifuged, washed with Dulbecco's PBS and stained with propidium iodide. The percentage of the sub-$G_1$ fraction consisting of apoptotic nuclei was calculated by CellQuest software (Becton Dickinson, CA, USA).

### Detection of CD95 expression

For flow cytometric analysis of CD95 expression, glioma cells were detached from the culture dishes, harvested into ice-cold complete medium containing 10% FCS, centrifuged and resuspended in FACS buffer (1% bovine serum albumin (BSA)/PBS/0.01% sodium azide). Subsequently, 10 μl of FITC-UB2 anti-Fas antibody was added per sample ($10^6$ cells) or, as a control, FITC-mouse $IgG_1$. After incubation and washing, samples were resuspended in 300 μl of PBS containing 1% formaldehyde and stored light-protected at 4°C before analysis by a Becton Dickinson FACScalibur cytometer. The specific fluorescence index (SFI) was calculated as the ratio of the mean fluorescence values obtained with the specific CD95 antibody and the isotype control antibody (Weller et al, 1995*c*).

**Table 1** $EC_{50}$ concentrations for cytotoxicity and growth inhibition of LN-229 and T98G human malignant glioma cells after exposure to taxol, CD95 ligand or TNF-α

| | Cytotoxicity | | Growth inhibition | |
|---|---|---|---|---|
| | LN-229 | T98G | LN-229 | T98G |
| Taxol (nM) | 38 (± 2) | 12 (± 1) | 82 (± 3) | 16 (± 2) |
| CD95 ligand (U $ml)^{-1}$ | 31 (± 3) | 9 (± 1) | 550 (± 13) | 25 (± 2) |
| TNF-α (ng $ml)^{-1}$ | > 250 | > 250 | > 250 | > 250 |

For cytotoxicity assays, the glioma cells were exposed for 72 h to taxol or the cytokines and viability was determined by crystal violet staining immediately thereafter. For growth inhibition assays, the cells were exposed to the agents for 24 h, had their medium replaced by drug-free medium and were allowed to recover for five generation times before crystal violet staining ($n$ = 3, mean ± s.d.)

© Cancer Research Campaign 1998

### Immunoblot analysis

Immunoblot studies for the detection of p53, p21, bcl-2 and bax protein expression were performed according to standard procedures as previously described (Weller et al, 1994). Briefly, glioma cell cultures treated as indicated were rinsed with cold PBS and harvested into cold PBS containing phenylmethylsulphonyl fluoride (10 µg ml$^{-1}$) with a cell scraper. The cells were centrifuged and resuspended in lysis buffer (0.12 M sodium chloride, 0.01 M Tris HCI, 0.005 M EDTA, 0.5% Triton, 2 µg ml$^{-1}$ aprotinin, 100 µg ml$^{-1}$ phenylmethylsulphonyl fluoride) for 10 min on ice. The lysates were centrifuged for 10 min in a microfuge at 13 000 r.p.m. Soluble supernatant protein (20 µg per lane) was separated on 10% (p53) or 15% (p21, bax, bcl-2) polyacrylamide gels and blotted onto nitrocellulose by standard procedures. The membranes were washed, incubated with primary antibody (2 µg ml$^{-1}$), washed, incubated with secondary antibody (alkaline phosphatase conjugate) and stained with nitroblue tetrazolium chloride and 5-bromo-4-chloro-3-indolyl phosphate in 0.1 M Tris HCl containing 50 mM magnesium chloride and 10 mM sodium chloride.

### Immunoprecipitation

Approximately $10^6$ human glioma cells were washed with ice-cold PBS, harvested into PBS containing phenylmethylsulphonyl fluoride (10 µg ml$^{-1}$) and lysed in lysis buffer for 10 min on ice (see above). Cell nuclei and debris were removed by centrifugation at 13 000 r.p.m. for 10 min. After pre-clearing the lysates with mouse IgG and protein G-Sepharose for 30 min and centrifugation, the lysates were incubated with bcl-2 antibodies overnight. Immunoprecipitates were then captured with protein G-Sepharose for 60 min. After washing the immunoprecipitates in lysis buffer, the samples were separated on a 15% polyacrylamide gel, blotted onto nitrocellulose, washed, incubated with biotinylated phosphoserine antibody and stained with nitroblue tetrazolium chloride and 5-bromo-4-chloro-3-indolyl phosphate in 0.1 M Tris HCl containing 50 mM magnesium chloride and 10 mM sodium chloride.

### Analysis of synergy

Synergy was evaluated (1) by the fractional product method of Webb (1963), which allows an evaluation of synergy at a defined level of effect, and (2) by the isobologram method (Berenbaum, 1981), which reveals synergy over a broad range of concentrations. In the fractional product method, the (additive) effect of two independently acting agents is defined as the product of the unaffected fractions after treatment with either agent alone: $f_u(1,2) = f_u(1) \times f_u(2)$. This formula allows the calculation of the predicted (additive) effect of co-treatment, based on the assumption that two agents do not interact or cooperate in inducing their effects. If the unaffected fraction, i.e. the relative percentage of surviving cells compared with untreated control cells, is below the calculated product $f_u(1,2)$ after co-treatment with two drugs, then synergy is assumed. Results are considered significant if by Student's *t*-test *P*-values are less than 0.05. Synergy was also calculated using the isobole analysis of Berenbaum (1981). The cells are exposed to different concentrations of each drug alone and of the two agents in combination. After treatment the relative survival compared with an untreated control is assessed by a viability assay. The next step is to plot the measured data as concentration effect curves. The $EC_{50}$ values of co-treatment are determined for each curve and

**Figure 1** Synergy of taxol and CD95 ligand, but not taxol and TNF-α: the fractional product method. The glioma cells were co-exposed for 24 h to taxol and CD95 ligand (**A–D**) or taxol and TNF-α (**E–H**) and allowed to recover for five generation times in fresh agent-free medium. The first bar in each panel shows the growth-inhibitory effect of CD95 ligand (**A–D**) or TNF-α (**E–H**) alone. The second bar shows the growth inhibitory effect of taxol alone. The third (open) bar shows the predicted independent (additive) effect of co-treatment (see Materials and methods). The fourth, black bar shows the observed effect of co-treatment. If the observed effect exceeds the predicted effect significantly, synergy is assumed ($n = 3$, mean and s.d., $*P < 0.05$, Student's *t*-test, observed effect compared with predicted effect)

© Cancer Research Campaign 1998

**Figure 2** Synergy of taxol and CD95 ligand: isobologram analysis. LN-229 and T98G cells were exposed to taxol, CD95 ligand or both for 72 h. Viability was assessed by crystal violet assay. Data are expressed as outlined in Materials and methods and are representative of three experiments with similar results. According to the isobole method of Berenbaum (1981), the straight line connecting the $EC_{50}$ values of two agents with monotherapy represents additivity (independence). Curves below this line indicate synergy; curves above this line indicate antagonism

**Table 2** Flow cytometric analysis of CD95 expression after exposure to taxol

| | Control | Taxol (50 nM) | Taxol (500 nM) | Taxol (1000 nM) |
|---|---|---|---|---|
| LN-229 | 2.08 | 1.83 | 2.11 | 2.19 |
| T98G | 1.82 | 1.69 | 1.49 | 1.49 |

LN-229 or T98G human malignant glioma cells were exposed to various concentrations of taxol for 24 h. CD95 expression was detected by flow cytometry as outlined in Materials and methods. Data are shown as SFI values, which represent the ratio of mean fluorescence values of CD95 antibody to isotype control antibody (see text).

divided by the $EC_{50}$ values for each drug in the absence of the other drug. The resulting data constitute the isobologram. The straight line connecting the $EC_{50}$ values of the two agents when applied alone represents additivity (independence). Points below this line indicate synergy; points above this line indicate antagonism.

**Figure 3** Cell cycle analysis of human malignant glioma cells exposed to taxol. LN-229 or T98G cells were untreated or exposed to taxol at 50 or 500 nM for 24 h in complete medium before cell cycle analysis (see Materials and methods). The first peak represents cells in $G_0/G_1$ phase, the second peak cells in $G_2/M$

### RESULTS

### Sensitivity of LN-229 and T98G human malignant glioma cells to taxol, CD95 ligand and TNF-α

The purpose of the present study was to examine a possible synergy of taxol-induced cytotoxicity with CD95 ligand-induced apoptosis. In the first set of experiments, we established concentration–response curves of sensitivity to taxol, CD95 ligand and TNF-α for LN-229 and T98G human malignant glioma cells in acute cytotoxicity and growth inhibition assays. For acute cytotoxicity assays, we exposed the glioma cells to these agents for 72 h and assessed viability by crystal violet staining or trypan blue exclusion. For growth inhibition assays, we exposed the cells for 24 h, washed them and allowed them to recover for five generation times, i.e. 5–7 days, before crystal violet staining. $EC_{50}$ concentrations were determined by linear regression (Table 1).

Taxol exhibited strong acute cytotoxic effects. The $EC_{50}$ concentrations in the crystal violet assay were 12 ± 1 nM for T98G and 38 ± 2 nM for LN-229 cells. The data obtained with the crystal violet assay were corroborated by trypan blue exclusion assays, as formally the crystal violet assay cannot differentiate cytotoxicity from growth inhibition. These two assays yield very similar results in the 72 h continuous exposure paradigm (data not shown). Glioma cells exposed to taxol showed classical signs of apoptosis, including membrane blebbing, condensation and segregation of chromatin into sharply delineated masses with apoptotic body formation, and condensation of the cytoplasm. Interestingly, we did not confirm the previously reported threshold for taxol cytotoxicity that had been interpreted as being indicative of a saturatable microtubular target (Helson et al, 1993; Silbergeld et al, 1995). Although a minor percentage of cells was still alive after a 24 h exposure, even to the highest concentrations of taxol examined, prolonged exposure to taxol was sufficient to kill 100% of the cells at 72 h, as confirmed by trypan blue exclusion. The growth inhibition assays also revealed prominent antiproliferative activity of taxol at nanomolar concentrations (Table 1).

We have previously reported that LN-229 and T98G cells are largely resistant to the proapoptotic effects of agonistic CD95 antibodies unless co-treated with inhibitors of RNA and protein synthesis, such as actinomycin D or cycloheximide (Weller et al,

©Cancer Research Campaign 1998

**Figure 4** Quantification of synergistic cytotoxicity by flow cytometry. T98G cells were exposed to taxol, CD95 ligand or both for 24 h. The cells were stained with propidium iodide and the sub-$G_1$ fraction of each sample was analysed by flow cytometry (see Materials and methods)

**Figure 5** p53, p21, bax and bcl-2 expression in glioma cells exposed to taxol and CD95 ligand. LN-229 (upper panel) or T98G (lower panel) cells were either untreated or exposed to taxol at 50, 500 or 1000 nM, in the absence (**A–D**) or presence (**E–H**) of CD95 ligand (80 U $ml^{-1}$ for LN-229, 8 U $ml^{-1}$ for T98G) for 24 h. Supernatant from *neo* vector control cells lacking CD95 ligand was used as control (**A–D**). Soluble protein lysates were prepared as described and 20 μg per lane was subjected to SDS-PAGE and immunoblot analysis (Weller et al, 1994; see Materials and methods)

© Cancer Research Campaign 1998

**Figure 6** Bcl-2 phosphorylation on serine after exposure of glioma cells to taxol. LN-229 glioma cells were exposed to 50 nM taxol for 24 h (**A, C**) or untreated (**B, D**). As outlined in Materials and methods, bcl-2 was immunoprecipitated and the precipitates subsequently subjected to SDS-PAGE and immunoblot analysis for phosphoserine residues. As control, the lysates of treated (**C**) and untreated (**D**) cells were immunoprecipitated with mouse IgG isotype control antibody and subjected to the same immunoblot procedure as the bcl-2 immunoprecipitates (**A, B**)

1994). Using the natural CD95 ligand derived from CD95 ligand-expressing murine neuroblastoma cells (Rensing-Ehl et al, 1995), efficient killing was achieved in the absence of such agents, LN-229 being more resistant to CD95-mediated apoptosis, as previously reported (Weller et al, 1994). Exposure to high concentrations of CD95 ligand, e.g. 800 U $ml^{-1}$ induced prominent cytotoxicity within 24 h, i.e. 32% in LN-229 and 83% in T98G cells. Accordingly, lower concentrations of CD95 ligand were selected for the synergy studies.

As previously reported (Zuber et al, 1988) and confirmed in our previous studies (Weller et al, 1994), relevant concentrations (< 250 ng $ml^{-1}$) of TNF-α were neither cytotoxic nor growth inhibitory to human glioma cells in the absence of inhibitors of RNA and protein synthesis (Table 1).

### Synergistic cytotoxicity and growth inhibition after co-exposure to taxol and CD95 ligand

Next, we asked whether cytotoxicity and growth inhibition induced by co-treatment with taxol and either CD95 ligand or TNF-α are synergistic, independent (additive) or antagonistic compared with the effects of exposure to either agent alone. The cells were treated with taxol or the cytokines, or their combination, either for 72 h or for 24 h with a recovery of five generation times. According to the fractional product method, there was synergy between taxol and CD95 ligand both in the growth inhibition assay (Figure 1) and in the cytotoxicity assay (data not shown) for both cell lines. In contrast, there was no such effect with the combination of taxol and TNF-α. This approach assumes synergy if the observed effect of co-treatment significantly exceeds the predicted (additive) effect. Synergy of taxol and CD95 ligand was also demonstrated by isobologram analysis (Figure 2). Here, the cells were exposed to broad concentration ranges of taxol, CD95 ligand or a combination of both for 72 h. Median effect concentrations were determined ($EC_{50}$) and depicted graphically as isobolograms, as outlined in Materials and methods. The discrepancy between the predicted and the observed effect allows visualization of the synergy. Synergy according to isobologram analysis was also confirmed in the growth inhibition paradigm (data not shown).

### Taxol does not enhance CD95 cell surface expression in human glioma cells

One possible mechanism of synergy between CD95 ligand and taxol is drug-induced augmentation of CD95 expression in the glioma cells, which would result in enhanced CD95-dependent signalling. Therefore, we examined CD95 expression in LN-229 and T98G glioma cells by flow cytometry after exposure to increasing concentrations of taxol for 24 h. Table 2 provides SFI values for CD95 expression (see Materials and methods). A SFI of 1.0 indicates that there is no difference in binding of CD95 antibody compared with an isotype control antibody. Untreated LN-229 and T98G human glioma cells were CD95 positive (SFI > 1.0) as previously reported (Weller et al, 1995*c*). Flow cytometry histograms revealed a rather homogeneous expression of CD95 (data not shown). This base line expression of CD95 was not enhanced by taxol. In fact, CD95 expression was unaffected in LN-229 cells and decreased rather than enhanced in T98G cells exposed to taxol.

### Independence of synergy from taxol-induced cell cycle arrest

To address the mechanism underlying synergy of taxol and CD95 ligand, we compared the effects of taxol (0, 50, 500 or 1000 nM) and taxol plus CD95 ligand (8 or 80 U $ml^{-1}$) on the cell cycle distribution of LN-229 and T98G cells. High concentrations of taxol (500 nM) induced a cell cycle block in $G_2$/M phase, whereas lower nanomolar concentrations had no such effect (Figure 3). In contrast, these lower concentrations were rather effective in inducing synergistic glioma cell killing and growth inhibition when co-administered with CD95 ligand (Figures 1, 2 and 4). This was further confirmed by flow cytometry, which was used to detect apoptotic cells as the sub-$G_1$ fraction that encompasses apoptotic cell nuclei and larger cellular remnants. The sub-$G_1$ fractions of T98G cells after a 24-h treatment with taxol only or CD95 ligand only were 1.5-fold or 1.7-fold higher than the sub-$G_1$ fraction of untreated control cells (Figure 4). However, the sub-$G_1$ fraction of glioma cells co-exposed to taxol and CD95 ligand was 5.1-fold increased. Absolute percentages of the sub-$G_1$ fraction were 4.1% in untreated cells, 6.0% with 50 nM taxol, 7.1% with 8 U $ml^{-1}$ CD95 ligand and 21.2% for 50 nM taxol plus 8 U $ml^{-1}$ CD95 ligand.

### p53 and p53 target gene expression after exposure of human glioma cells to taxol and CD95 ligand: taxol-induced phosphorylation of the bcl-2 protein

LN-229 has wild-type p53 activity, whereas T98G is mutant for p53 (Van Meir et al, 1994; Weller et al, 1997). While the p53 wild-type cell line LN-229 was more resistant to taxol than the p53 mutant cell line T98G, this difference was not striking (Table 1). We examined changes in p53 expression and expression of two p53 target genes, *p21* and *bax*, after exposure to taxol and asked whether such changes were modulated during synergistic augmentation of taxol toxicity by CD95 ligand (Figure 5). Excessive concentrations of taxol (1000 nM) were required to induce p53 expression in LN-229 cells (upper panel, lane D). This p53 induction was not modulated by co-exposure to CD95 ligand (lane H). In contrast, no change in p53 expression was observed in T98G cells, as expected for a p53 mutant cell line. Note that most p53

© Cancer Research Campaign 1998

mutations result in accumulation of p53 protein because of a prolonged half-life of the mutant protein, as is shown here for T98G. The p21 protein is thought to be responsible for p53- and drug-induced growth arrest in $G_0/G_1$. Interestingly, there was no induction of p21 in either cell line, even though p53 was induced in LN-229 cells. Camptothecin (5 μM) was used as a positive control to illustrate that p21 can be induced in these glioma cell lines (p21 blots, right outer lanes). These data fit nicely with the flow cytometry findings, which clearly indicated a $G_2/M$, but not $G_0/G_1$, arrest after glioma cell exposure to taxol (Figure 3).

Further, we assessed expression of the bax protein, a key mediator of p53-induced apoptosis as well as p53-independent cell death, which is antagonized by the prototype anti-apoptotic protein bcl-2. Again, there was no modulation of bax expression by either taxol alone or taxol plus CD95 ligand, suggesting that (1) taxol-induced p53 activity is insufficient for significant transcriptional effects on major target genes and that (2) enhanced bax expression mediates neither taxol- nor CD95 ligand-induced apoptosis. However, susceptibility to apoptosis may depend more on the relative expression levels of functional antagonists, such as bax and bcl-2 or bcl-x, than on their absolute levels. To inhibit the proapoptotic effects of bax homodimers, bcl-2 forms heterodimers with bax (Oltvai et al, 1993). Recently, it has also been shown that taxol induces phosphorylation of bcl-2 and that phosphorylated bcl-2 fails to prevent apoptosis because of its inability to bind Bax (Haldar et al, 1995, 1996). In line with these data, we observed a mobility shift of the bcl-2 protein in glioma cells exposed to taxol (Figure 5), which is suggestive of phosphorylation (Haldar et al, 1995). Immunoprecipitation of bcl-2 and subsequent immunoblot analysis of phosphoserine revealed that the observed change in mobility of bcl-2 was due to phosphorylation on serine (Figure 6). In contrast to p53 activation and $G_2/M$ cell cycle arrest, bcl-2 phosphorylation was seen even with low concentrations of taxol (50 nM), which are sufficient for synergy with CD95 ligand-induced cytotoxicity. CD95 ligand administered alone had no such effect and did not modulate the effect of taxol on bcl-2 migration patterns upon co-treatment. In fact, even excessive, highly cytotoxic concentrations of CD95 ligand did not induce specific changes in the candidate regulatory proteins for apoptosis examined here (data not shown).

### DISCUSSION

Human malignant glioma cells are rather resistant to multiple proapoptotic stimuli, including irradiation and most cytotoxic drugs. The mechanisms underlying this failure to respond to therapy may include loss of p53 and enhanced expression of anti-apoptotic gene products, such as bcl-2, but are incompletely understood (Weller, 1995). We have previously reported that glioma cells are not resistant to apoptosis induced by agonistic antibodies that activate the CD95 cytokine receptor protein (Weller et al, 1994). Moreover, we have noted that these cells are even more sensitive to apoptosis induced by the endogenous CD95 ligand, a cytokine homologous to TNF-α (Roth et al, 1997). The main problem with CD95 ligand-based immunotherapy of malignant gliomas is probably prevention of systemic toxicity as agonistic antibodies to CD95 induce liver failure within a few hours when applied systemically (Ogasawara et al, 1993). However, a local CD95-based approach to malignant glioma appears to be feasible. Thus, growth of an intraperitoneal lymphoma can be controlled by intraperitoneal CD95 ligand applied locally (Rensing-Ehl et al, 1995), and intra-articular application of CD95 antibodies can control arthritis (Fujisawa et al, 1996), both in the absence of systemic toxicity. Further, circulation of CD95 ligand is not inevitably lethal (Sato et al, 1996). As systemic toxicity depends on the dose of CD95 ligand applied and as maximal glioma cell killing is to be achieved, we have been particularly interested in immunochemotherapy based on co-treatment of glioma cells with CD95 ligand and cancer chemotherapeutic drugs (Roth et al, 1997). The current focus is on taxol for several reasons: (1) taxol has previously been reported (Cahan et al, 1994; Silbergeld et al, 1995) and confirmed here to have strong anti-glioma effects in vitro; (2) taxol limits growth of glioma xenografts in mice (Riondel et al, 1992); and (3) taxol has already been adapted for local controlled release therapy of malignant gliomas and shown to be effective in a rat glioma model (Walter et al, 1994).

The present study shows that the combination of taxol and CD95 ligand results in synergistic cytotoxicity and growth inhibition of human malignant glioma cells. Of note, no such effect is seen with TNF-α, a cytokine previously reported to cooperate with taxol in inducing cytotoxicity of ovarian carcinoma cells (Williams et al, 1992; Berkova and Pagé, 1995). Synergy was not due to an enhanced expression of CD95 in taxol-treated glioma cells (Table 2). Neither $G_2/M$ arrest nor induced expression of p53 or p21 was required for taxol toxicity of the glioma cells (Figures 3 and 5). Further, neither cell cycle effects nor constitutive or induced expression of these gene products appeared to be involved in the mechanisms underlying synergy of taxol and CD95 ligand, because synergy was obtained at concentrations of taxol that were insufficient to induce $G_2/M$ arrest or p53 expression. In contrast, positive interactions of taxol and irradiation appear to involve enhanced expression of p53 and p21 (Tishler and Lamppu, 1996). The failure of taxol to induce p21 expression in the glioma cells examined here is consistent with a $G_2/M$ arrest as p21 is a mediator of $G_0/G_1$ arrest (Waldmann et al, 1995). Yet, taxol effects are probably cell type-specific as p21 was strongly induced in prostate and breast carcinoma cell lines (Blagosklonny et al, 1996).

We confirm that taxol induces phosphorylation of bcl-2 (Haldar, 1995). In contrast to p53 induction and $G_2/M$ cell cycle effects, the phosphorylation of bcl-2 protein was seen at low taxol concentrations that were sufficient to induce synergistic cytotoxicity of taxol and CD95 ligand. Phosphorylation of bcl-2 inhibits its heterodimerization with bax. Thus, although bax was not induced by taxol (Figure 5), the free levels of bax are likely to increase after taxol treatment because less bcl-2 is available for complex formation with bax (Blagosklonny et al, 1996; Haldar et al, 1996). The fact that enhanced levels of free bax in LN-229 and T98G cells are associated with increased susceptibility to CD95 ligand is in perfect agreement with our previous observation that ectopic expression of bcl-2 in these cells attenuates the cytotoxic effects of agonistic CD95 antibodies (Weller et al, 1995*b*) and natural CD95 ligand (Roth et al, 1997).

CD95 ligand-based proapoptotic immunotherapy of malignant glioma is a promising novel option for the management of malignant gliomas (Weller et al, 1994, 1995*a–c*), which appears to circumvent the inherent resistance of these neoplasms to several other stimuli for apoptotic cell death. Here, we have demonstrated synergy of CD95 ligand and taxol against human malignant glioma cells in vitro. Synergy was obtained at concentrations of taxol (50–100 nM) that are achieved in plasma 24 h after intravenous

© Cancer Research Campaign 1998

taxol (Glantz et al, 1995*b*). For reasons outlined above, both CD95 ligand and taxol are likely to be maximally effective when administered using a locoregionary approach. The present results call for an evaluation of local CD95 ligand plus taxol in an animal model of glioma.

### ACKNOWLEDGEMENT

This study was supported by the Deutsche Forschungsgemeinschaft (We 1502/3-1).

### REFERENCES

Berenbaum MC (1981) Criteria for analyzing interactions between biologically active agents. *Adv Cancer Res* **35**: 269–335

Berkova N and Pagé M (1995) Addition of hTNF-alpha potentiates cytotoxicity of Taxol in human ovarian cancer lines. *Anticancer Res* **15**: 863–866

Bhalla K, Ibrado AM, Tourkina E, Tang C, Maloney ME and Huang Y (1993) Taxol induces internucleosomal DNA fragmentation associated with programmed cell death in human myeloid leukemia cells. *Leukemia* **7**: 563–568

Blagosklonny MV, Schulte TW, Nguyen P, Mimnaugh EG, Trepel J and Neckers L (1995) Taxol induction of p21 waf1 and p53 requires c-raf-1. *Cancer Res* **55**: 4623–4626

Blagosklonny MV, Schulte T, Nguyen P, Trepel J and Neckers LM (1996) Taxol-induced apoptosis and phosphorylation of Bcl-2 protein involves c-Raf-1 and represents a novel c-Raf-1 signal transduction pathway. *Cancer Res* **56**: 1851–1854

Cahan MA, Walter KA, Colvin OM and Brem H (1994) Cytotoxicity of taxol in vitro against human and rat malignant brain tumors. *Cancer Chemother Pharmacol* **33**: 441–444

Chamberlain MC and Kormanik P (1995) Salvage chemotherapy with paclitaxel for recurrent primary brain tumors. *J Clin Oncol* **13**: 2066–2071

Ding AH, Porteu F, Sanchez E and Nathan CF (1990) Shared actions of endotoxin and taxol on TNF receptors and TNF release. *Science* **248**: 370–372

Fujisawa K, Asahara H, Okamoto K, Aono H, Hasunuma T, Kobata T, Iwakura Y, Yonehara S, Sumida T and Nishioka K (1996) Therapeutic effect of the anti-fas antibody on arthritis in HTLV-I tax transgenic mice. *J Clin Invest* **98**: 271–278

Glantz MJ, Choy H, Kearns CM, Akerley W and Egorin MJ (1995*a*) Weekly, outpatient paclitaxel and concurrent cranial irradiation in adults with brain tumors: preliminary results and promising directions. *Semin Oncol* **22**(suppl. 12): 26–32

Glantz MJ, Choy H, Kearns CM, Mills PC, Wahlberg LU, Zuhowski EG, Calabresi P and Egorin MJ (1995*b*) Paclitaxel disposition in plasma and central nervous systems of humans and rats with brain tumors. *J Natl Cancer Inst* **87**: 1077–1081

Haldar S, Jena N and Croce CM (1995) Inactivation of Bcl-2 by phosphorylation. *Proc Natl Acad Sci USA* **92**: 4507–4511

Haldar S, Chintapalli J and Croce CM (1996) Taxol induces bcl-2 phosphorylation and death of prostate cancer cells. *Cancer Res* **56**: 1253–1255

Heimans JJ, Vermorken JB, Wolbers JG, Eeltink CM, Meijer OWM, Taphoorn MJB and Beijnen JH (1994) Paclitaxel (taxol) concentrations in brain tumor tissue. *Ann Oncol* **5**: 951–953

Helson L, Helson C, Malik S, Ainsworth S and Mangiardi J (1993) A saturation threshold for taxol cytotoxicity in human glial and neuroblastoma cells. *Anticancer Drug* **4**: 487–490

Horwitz SB, Lothstein L, Manfredi J, Mellado W, Parness J, Roy SN, Schiff PB, Sorbara L and Zeheb R (1986) Taxol: mechanisms of action and resistance. *Ann NY Acad Sci* **466**: 733–744

Milross CG, Mason KA, Hunter NR, Chung WK, Peters LJ and Milas L (1996) Relationship of mitotic arrest and apoptosis to antitumor effect of paclitaxel. *J Natl Cancer Inst* **88**: 1308–1314

Ogasawara J, Watanabe-Fukunaga R, Adachi M, Matsuzawa A, Kasugai T, Kitamura Y, Itoh N, Suda T and Nagata S (1993) Lethal effect of the anti-Fas antibody in mice. *Nature* **364**: 806–809

Oltvai ZN, Milliman CL and Korsmeyer SJ (1993) Bcl-2 heterodimerizes in vivo with a conserved homolog, Bax, that accelerates programmed cell death. *Cell* **74**: 609–619

Prados MD, Schold SC, Spence AM, Berger MS, McAllister LD, Mehta MP, Gilbert R, Fulton D, Kuhn J, Lamborn K, Rector DJ and Chang SM (1996) Phase II study of paclitaxel in patients with recurrent malignant glioma. *J Clin Oncol* **14**: 2316–2321

Rensing-Ehl A, Frei K, Flury R, Matiba B, Mariani SM, Weller M, Aebischer P, Krammer PH and Fontana A (1995) Loco-regional Fas/APO-1 (CD95) ligand-mediated tumor cell killing in vivo. *Eur J Immunol* **25**: 2253–2258

Riondel J, Jacrot M, Fessi H, Puisieux F and Potier (1992) Effects of free and liposome-encapsulated taxol on two brain tumors xenografted into nude mice. *In Vivo* **6**: 23–27

Roth W, Fontana A, Trepel M, Reed JC, Dichgans J and Weller M (1997) Immunochemotherapy of malignant glioma: synergistic activity of CD95 ligand and chemotherapeutics. *Cancer Immunol Immunother* **44**: 55–63

Rowinsky EK, Donehower RC, Jones RJ and Tucker RW (1988) Microtubule changes and cytotoxicity in leukemic cell lines treated with taxol. *Cancer Res* **48**: 4093–4100

Sato K, Kimura F, Nakamura Y, Murakami H, Yoshida M, Tanaka M, Nagata S, Kanatani Y, Wakimoto N, Nagata N and Motoyoshi K (1996) An aggressive nasal lymphoma accompanied by high levels of soluble Fas ligand. *Br J Haematol* **94**: 379–382

Schiff PB, Fant J and Horwitz SB (1979) Promotion of microtubule assembly in vitro by taxol. *Nature* **277**: 665–667

Silbergeld DL, Chicoine MR and Madsen CL (1995) In vitro assessment of Taxol for human glioblastoma: chemosensitivity and cellular locomotion. *Anticancer Drug* **6**: 270–276

Tishler RB and Lamppu DM (1996) The interaction of taxol and vinblastine with radiation induction of p53 and $p21^{waf1/cip1}$. *Br J Cancer* **74**(suppl. 27): S82–S85

Tishler RB, Geard CR, Hall EJ and Schiff PB (1992) Taxol sensitizes human astrocytoma cells to radiation. *Cancer Res* **52**: 3495–3497

Van Meir EG, Kikuchi T, Tada M, Li H, Diserens AC, Wojcik BE, Huang HJS, Friedmann T, De Tribolet N and Cavenee WK (1994) Analysis of the p53 gene and its expression in human glioblastoma cells. *Cancer Res* **54**: 649–652

Wahl AF, Donaldson KL, Fairchild C, Lee FYF, Foster SA, Demers GW and Galloway DA (1996) Loss of normal p53 function confers sensitization to Taxol by increasing G2/M arrest and apoptosis. *Nature Med* **2**: 72–79

Waldman T, Kinzler KW and Vogelstein B (1995) p21 is necessary for the p53-mediated G1 arrest in human cancer cells. *Cancer Res* **55**: 5187–5190

Walter KA, Cahan MA, Gur A, Tyler B, Hilton J, Colvin OM, Burger PC, Domb A and Brem H (1994) Interstitial taxol delivered from a biodegradable polymer implant against experimental malignant glioma. *Cancer Res* **54**: 2207–2212

Webb JL (1963) Effect of more than one inhibitor. In *Enzyme and Metabolic Inhibitors*, Vol. 1, pp. 66–79, 487–512. Academic Press: New York

Weller M (1996) Genetic regulation and therapeutic modulation of apoptosis in human malignant glioma. *Cell Physiol Biochem* **6**: 376–380

Weller M and Fontana A (1995) The failure of current immunotherapy for malignant glioma. Tumor-derived TGF-β, T cell apoptosis, and the immune privilege of the brain. *Brain Res Rev* **21**: 128–151

Weller M, Frei K, Groscurth P, Krammer PH, Yonekawa Y and Fontana A (1994) Anti-Fas/APO-1 antibody-mediated apoptosis of cultured human glioma cells. Induction and modulation of sensitivity by cytokines. *J Clin Invest* **94**: 954–964

Weller M, Frei K, Malipiero U, Groscurth P, Yonekawa Y, Krammer P and Fontana A (1995*a*) Fas/APO-1-mediated apoptosis of human malignant glioma. *Neurology* **45**(suppl. 4): A401, 859S

Weller M, Malipiero U, Aguzzi A, Reed JC and Fontana A (1995*b*). Protooncogene bcl-2 gene transfer abrogates Fas/APO-1 antibody-mediated apoptosis of human malignant glioma cells and confers resistance to chemotherapeutic drugs and therapeutic irradiation. *J Clin Invest* **95**: 2633–2643

Weller M, Malipiero U, Rensing-Ehl A, Barr P and Fontana A (1995*c*) Fas/APO-1 gene transfer for human malignant glioma. *Cancer Res* **55**: 2936–2944

Weller M, Trepel M, Grimmel C, Schabet M, Bremen D, Krajewski S and Reed JC (1997) Hypericin-induced apoptosis of human malignant glioma cells is light dependent, independent of bcl-2 expression and does not require wild type p53. *Neurol Res* **19**: 459–470

Williams S, Mutch DG, Xu L and Collins JL (1992) Divergent effects of taxol on tumor necrosis factor-alpha-mediated cytolysis of ovarian carcinoma cells. *Am J Obstet Gynecol* **167**: 1870–1876

Zuber P, Accolla RS, Carrel S, Diserens AC and De Tribolet N (1988) Effects of recombinant human tumor necrosis factor-alpha-α on the surface phenotype and the growth of human malignant glioma cell lines. *Int J Cancer* **42**: 780–786

© Cancer Research Campaign 1998


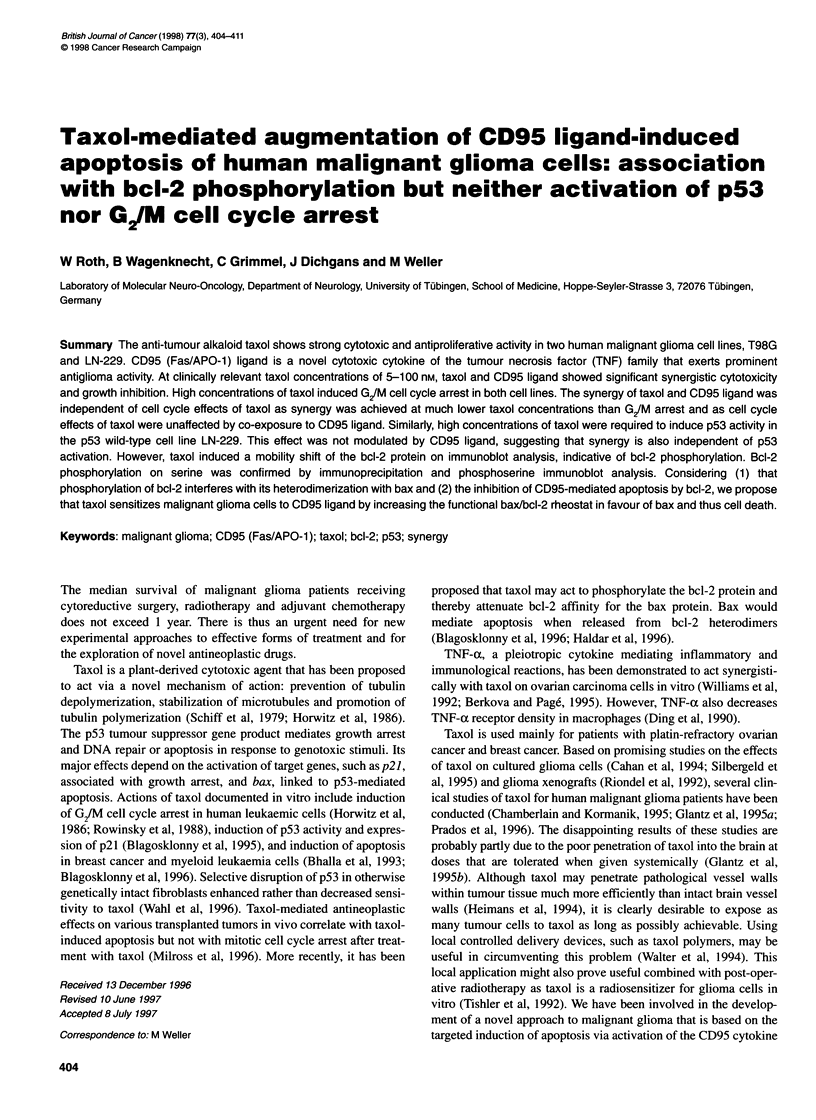

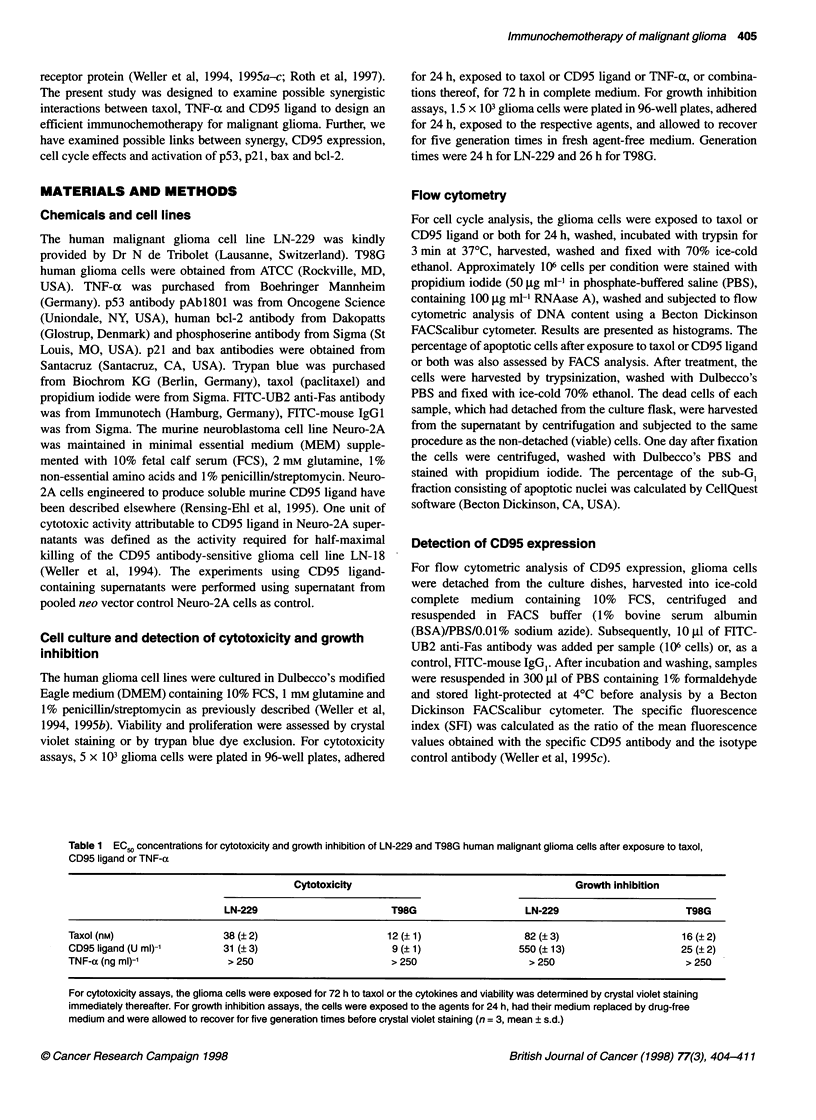

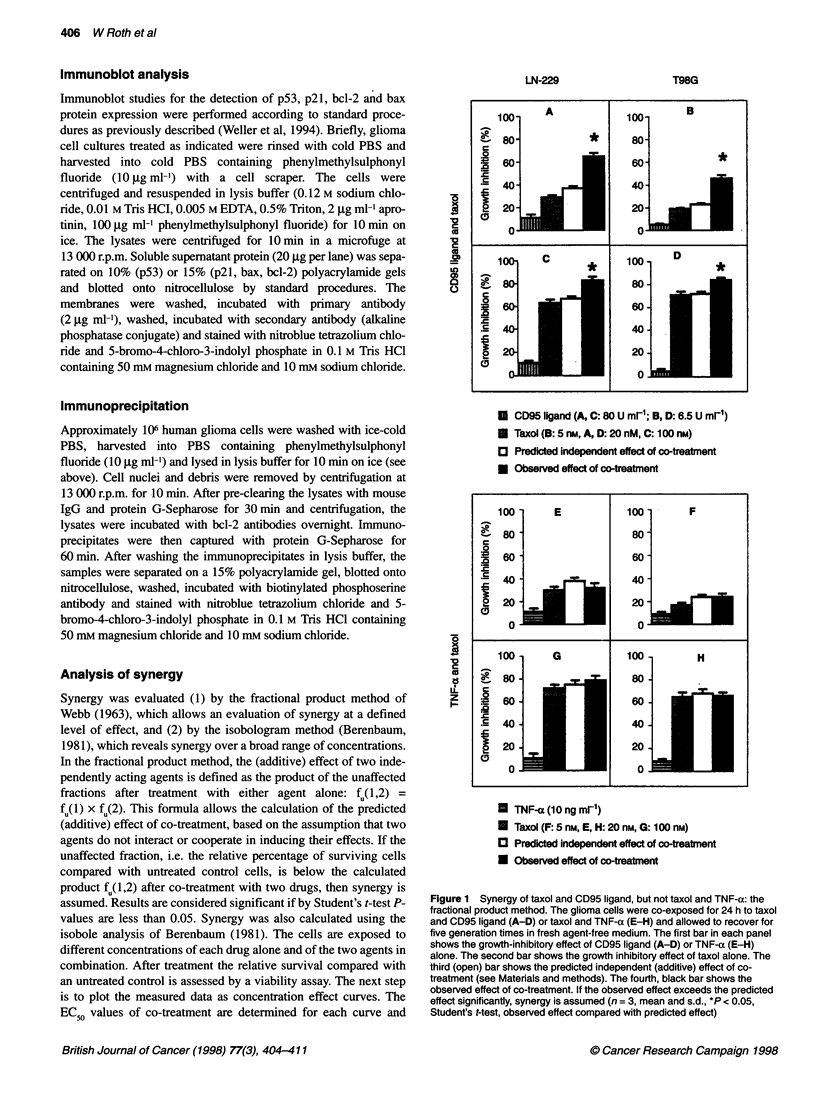

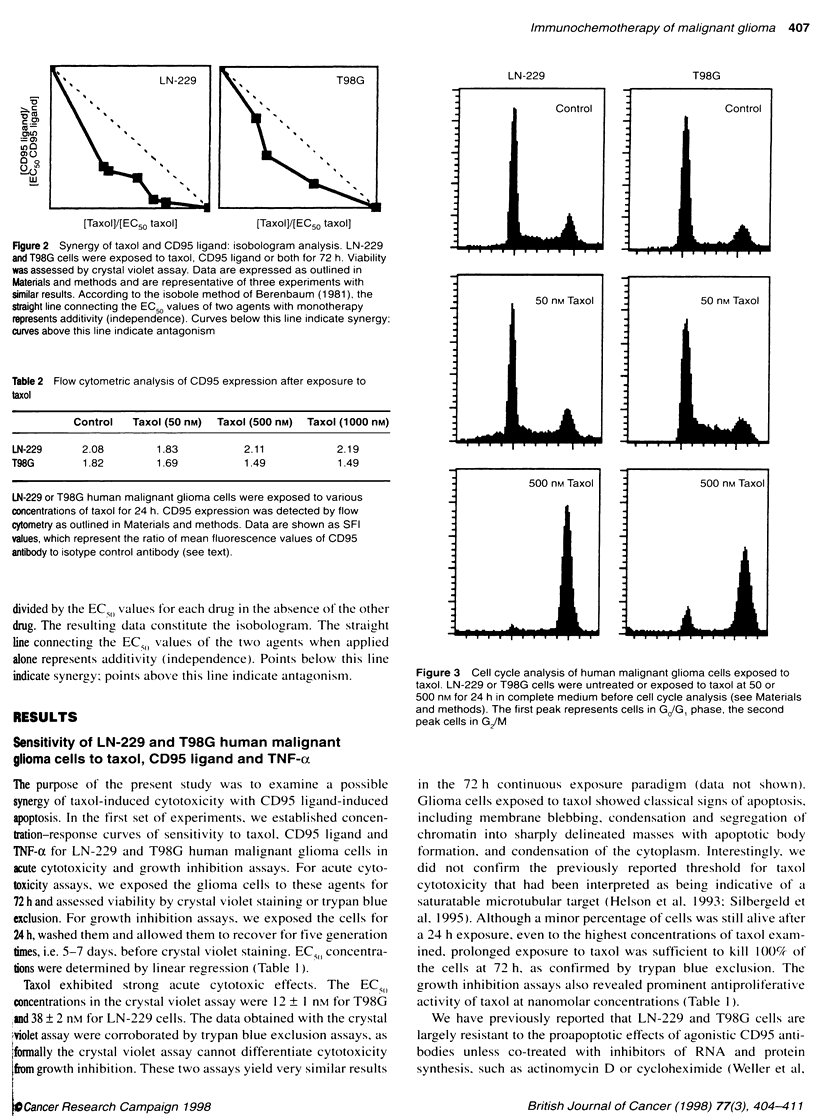

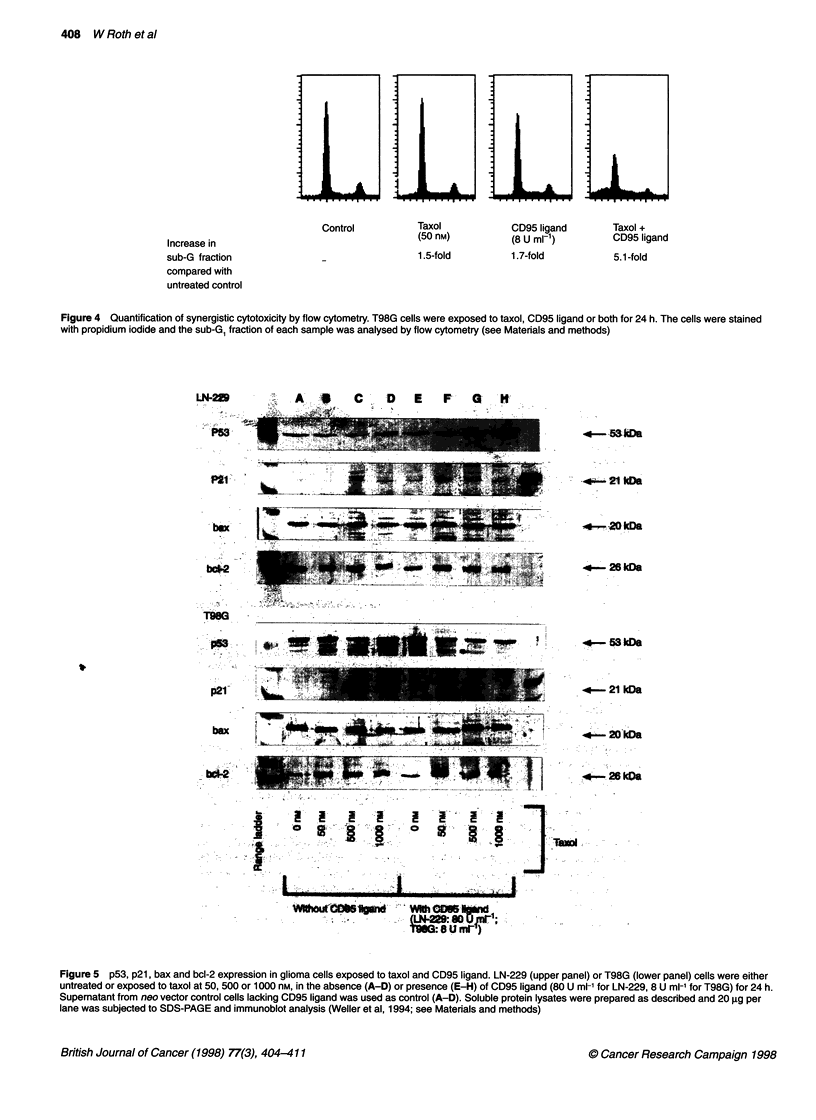

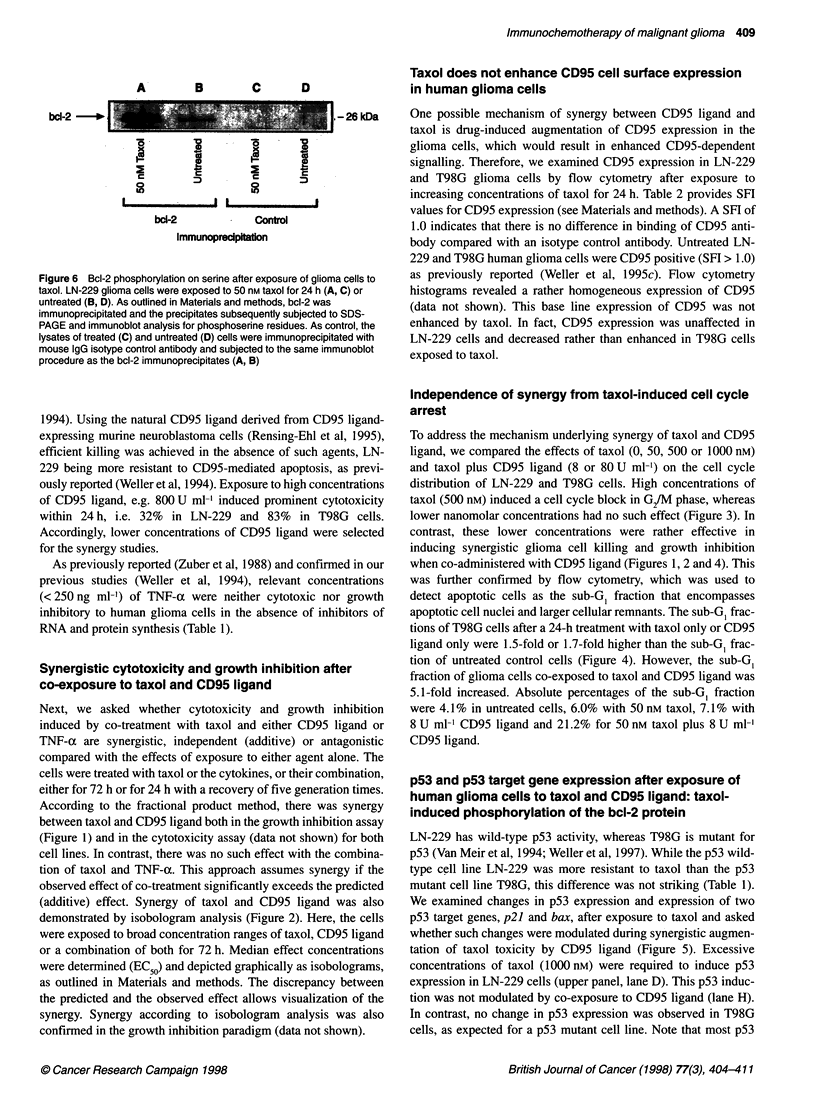

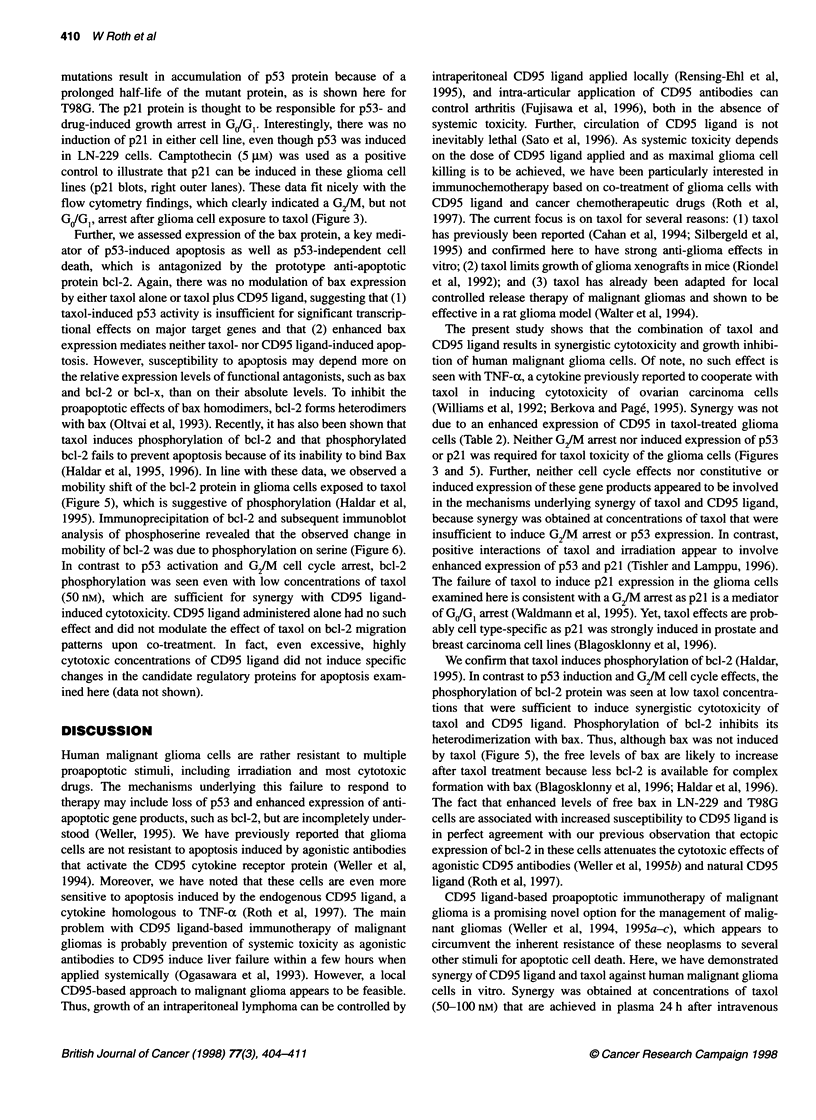

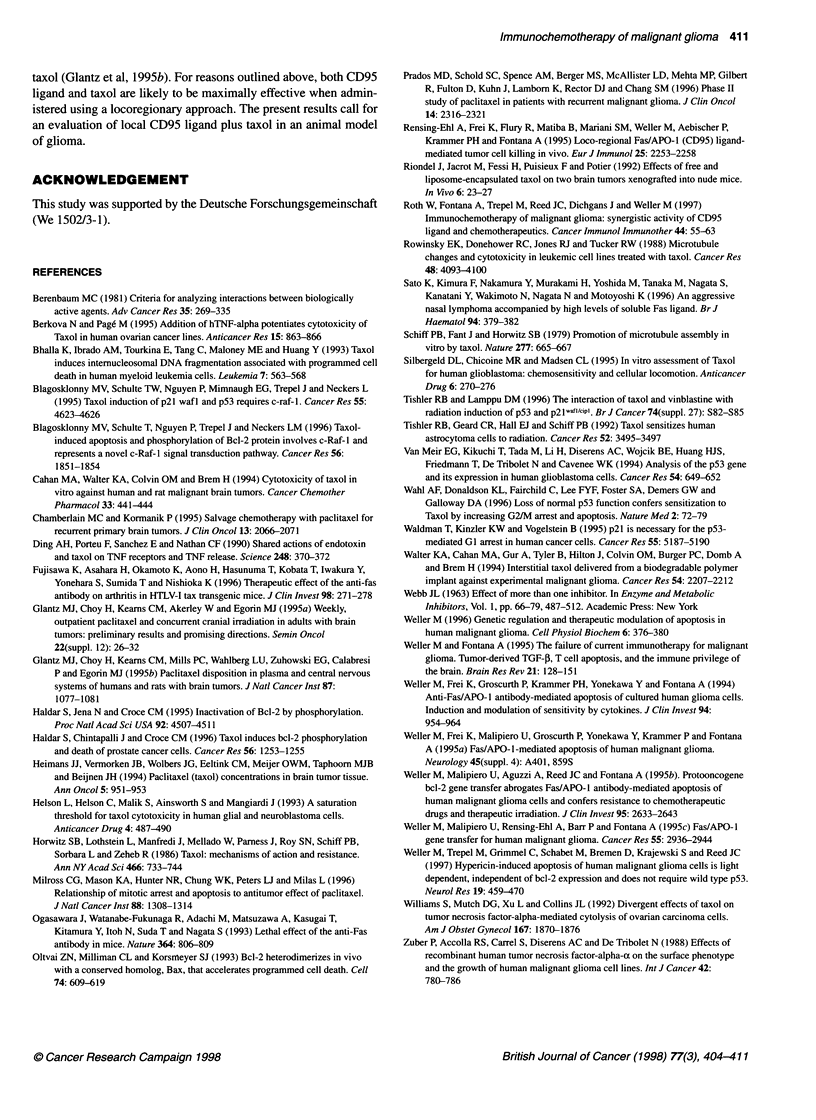


## References

[OCR_00808] Berenbaum M. C. (1981). Criteria for analyzing interactions between biologically active agents.. Adv Cancer Res.

[OCR_00812] Berkova N., Pagé M. (1995). Addition of hTNF alpha potentiates cytotoxicity of taxol in human ovarian cancer lines.. Anticancer Res.

[OCR_00816] Bhalla K., Ibrado A. M., Tourkina E., Tang C., Mahoney M. E., Huang Y. (1993). Taxol induces internucleosomal DNA fragmentation associated with programmed cell death in human myeloid leukemia cells.. Leukemia.

[OCR_00821] Blagosklonny M. V., Schulte T. W., Nguyen P., Mimnaugh E. G., Trepel J., Neckers L. (1995). Taxol induction of p21WAF1 and p53 requires c-raf-1.. Cancer Res.

[OCR_00826] Blagosklonny M. V., Schulte T., Nguyen P., Trepel J., Neckers L. M. (1996). Taxol-induced apoptosis and phosphorylation of Bcl-2 protein involves c-Raf-1 and represents a novel c-Raf-1 signal transduction pathway.. Cancer Res.

[OCR_00832] Cahan M. A., Walter K. A., Colvin O. M., Brem H. (1994). Cytotoxicity of taxol in vitro against human and rat malignant brain tumors.. Cancer Chemother Pharmacol.

[OCR_00837] Chamberlain M. C., Kormanik P. (1995). Salvage chemotherapy with paclitaxel for recurrent primary brain tumors.. J Clin Oncol.

[OCR_00841] Ding A. H., Porteu F., Sanchez E., Nathan C. F. (1990). Shared actions of endotoxin and taxol on TNF receptors and TNF release.. Science.

[OCR_00845] Fujisawa K., Asahara H., Okamoto K., Aono H., Hasunuma T., Kobata T., Iwakura Y., Yonehara S., Sumida T., Nishioka K. (1996). Therapeutic effect of the anti-Fas antibody on arthritis in HTLV-1 tax transgenic mice.. J Clin Invest.

[OCR_00849] Glantz M. J., Choy H., Kearns C. M., Akerley W., Egorin M. J. (1995). Weekly, outpatient paclitaxel and concurrent cranial irradiation in adults with brain tumors: preliminary results and promising directions.. Semin Oncol.

[OCR_00855] Glantz M. J., Choy H., Kearns C. M., Mills P. C., Wahlberg L. U., Zuhowski E. G., Calabresi P., Egorin M. J. (1995). Paclitaxel disposition in plasma and central nervous systems of humans and rats with brain tumors.. J Natl Cancer Inst.

[OCR_00865] Haldar S., Chintapalli J., Croce C. M. (1996). Taxol induces bcl-2 phosphorylation and death of prostate cancer cells.. Cancer Res.

[OCR_00861] Haldar S., Jena N., Croce C. M. (1995). Inactivation of Bcl-2 by phosphorylation.. Proc Natl Acad Sci U S A.

[OCR_00869] Heimans J. J., Vermorken J. B., Wolbers J. G., Eeltink C. M., Meijer O. W., Taphoorn M. J., Beijnen J. H. (1994). Paclitaxel (Taxol) concentrations in brain tumor tissue.. Ann Oncol.

[OCR_00874] Helson L., Helson C., Malik S., Ainsworth S., Mangiardi J. (1993). A saturation threshold for taxol cytotoxicity in human glial and neuroblastoma cells.. Anticancer Drugs.

[OCR_00879] Horwitz S. B., Lothstein L., Manfredi J. J., Mellado W., Parness J., Roy S. N., Schiff P. B., Sorbara L., Zeheb R. (1986). Taxol: mechanisms of action and resistance.. Ann N Y Acad Sci.

[OCR_00884] Milross C. G., Mason K. A., Hunter N. R., Chung W. K., Peters L. J., Milas L. (1996). Relationship of mitotic arrest and apoptosis to antitumor effect of paclitaxel.. J Natl Cancer Inst.

[OCR_00889] Ogasawara J., Watanabe-Fukunaga R., Adachi M., Matsuzawa A., Kasugai T., Kitamura Y., Itoh N., Suda T., Nagata S. (1993). Lethal effect of the anti-Fas antibody in mice.. Nature.

[OCR_00894] Oltvai Z. N., Milliman C. L., Korsmeyer S. J. (1993). Bcl-2 heterodimerizes in vivo with a conserved homolog, Bax, that accelerates programmed cell death.. Cell.

[OCR_00899] Prados M. D., Schold S. C., Spence A. M., Berger M. S., McAllister L. D., Mehta M. P., Gilbert M. R., Fulton D., Kuhn J., Lamborn K. (1996). Phase II study of paclitaxel in patients with recurrent malignant glioma.. J Clin Oncol.

[OCR_00905] Rensing-Ehl A., Frei K., Flury R., Matiba B., Mariani S. M., Weller M., Aebischer P., Krammer P. H., Fontana A. (1995). Local Fas/APO-1 (CD95) ligand-mediated tumor cell killing in vivo.. Eur J Immunol.

[OCR_00910] Riondel J., Jacrot M., Fessi H., Puisieux F., Potier (1992). Effects of free and liposome-encapsulated taxol on two brain tumors xenografted into nude mice.. In Vivo.

[OCR_00915] Roth W., Fontana A., Trepel M., Reed J. C., Dichgans J., Weller M. (1997). Immunochemotherapy of malignant glioma: synergistic activity of CD95 ligand and chemotherapeutics.. Cancer Immunol Immunother.

[OCR_00919] Rowinsky E. K., Donehower R. C., Jones R. J., Tucker R. W. (1988). Microtubule changes and cytotoxicity in leukemic cell lines treated with taxol.. Cancer Res.

[OCR_00924] Sato K., Kimura F., Nakamura Y., Murakami H., Yoshida M., Tanaka M., Nagata S., Kanatani Y., Wakimoto N., Nagata N. (1996). An aggressive nasal lymphoma accompanied by high levels of soluble Fas ligand.. Br J Haematol.

[OCR_00930] Schiff P. B., Fant J., Horwitz S. B. (1979). Promotion of microtubule assembly in vitro by taxol.. Nature.

[OCR_00934] Silbergeld D. L., Chicoine M. R., Madsen C. L. (1995). In vitro assessment of Taxol for human glioblastoma: chemosensitivity and cellular locomotion.. Anticancer Drugs.

[OCR_00942] Tishler R. B., Geard C. R., Hall E. J., Schiff P. B. (1992). Taxol sensitizes human astrocytoma cells to radiation.. Cancer Res.

[OCR_00939] Tishler R. B., Lamppu D. M. (1996). The interaction of taxol and vinblastine with radiation induction of p53 and p21 WAF1/CIP1.. Br J Cancer Suppl.

[OCR_00946] Van Meir E. G., Kikuchi T., Tada M., Li H., Diserens A. C., Wojcik B. E., Huang H. J., Friedmann T., de Tribolet N., Cavenee W. K. (1994). Analysis of the p53 gene and its expression in human glioblastoma cells.. Cancer Res.

[OCR_00950] Wahl A. F., Donaldson K. L., Fairchild C., Lee F. Y., Foster S. A., Demers G. W., Galloway D. A. (1996). Loss of normal p53 function confers sensitization to Taxol by increasing G2/M arrest and apoptosis.. Nat Med.

[OCR_00955] Waldman T., Kinzler K. W., Vogelstein B. (1995). p21 is necessary for the p53-mediated G1 arrest in human cancer cells.. Cancer Res.

[OCR_00959] Walter K. A., Cahan M. A., Gur A., Tyler B., Hilton J., Colvin O. M., Burger P. C., Domb A., Brem H. (1994). Interstitial taxol delivered from a biodegradable polymer implant against experimental malignant glioma.. Cancer Res.

[OCR_00971] Weller M., Fontana A. (1995). The failure of current immunotherapy for malignant glioma. Tumor-derived TGF-beta, T-cell apoptosis, and the immune privilege of the brain.. Brain Res Brain Res Rev.

[OCR_00976] Weller M., Frei K., Groscurth P., Krammer P. H., Yonekawa Y., Fontana A. (1994). Anti-Fas/APO-1 antibody-mediated apoptosis of cultured human glioma cells. Induction and modulation of sensitivity by cytokines.. J Clin Invest.

[OCR_00993] Weller M., Malipiero U., Rensing-Ehl A., Barr P. J., Fontana A. (1995). Fas/APO-1 gene transfer for human malignant glioma.. Cancer Res.

[OCR_00997] Weller M., Trepel M., Grimmel C., Schabet M., Bremen D., Krajewski S., Reed J. C. (1997). Hypericin-induced apoptosis of human malignant glioma cells is light-dependent, independent of bcl-2 expression, and does not require wild-type p53.. Neurol Res.

[OCR_01004] Williams S., Mutch D. G., Xu L., Collins J. L. (1992). Divergent effects of taxol on tumor necrosis factor-alpha-mediated cytolysis of ovarian carcinoma cells.. Am J Obstet Gynecol.

[OCR_01009] Zuber P., Accolla R. S., Carrel S., Diserens A. C., de Tribolet N. (1988). Effects of recombinant human tumor necrosis factor-alpha on the surface phenotype and the growth of human malignant glioma cell lines.. Int J Cancer.

